# Changes in acute hospital costs after employing clinical facilitators to improve stroke care in Victoria, Australia

**DOI:** 10.1186/s12913-018-3836-9

**Published:** 2019-01-18

**Authors:** Dominique A. Cadilhac, Helen M. Dewey, Sonia Denisenko, Christopher F. Bladin, Atte Meretoja

**Affiliations:** 1 Translational Public Health and Evaluation Division, Stroke and Ageing Research, Department of Medicine, School of Clinical Sciences at Monash Health, Monash University, Clayton, Australia; 2 Stroke Division, Florey Institute of Neuroscience and Mental Health, University of Melbourne, Heidelberg, Australia; 3 System Design, Planning & Decision Support Unit, Policy & Planning Branch, Department of Health and Human Services, Melbourne, Australia; 4 Eastern Health Clinical School, Monash University, Box Hill, Australia; 5 Department of Medicine, Royal Melbourne Hospital, University of Melbourne, Parkville, Australia; 6 Neurocenter, Helsinki University Hospital, Helsinki, Finland

**Keywords:** Stroke, Cost-benefit analysis, Healthcare, Policy

## Abstract

**Background:**

Hospital costs for stroke are increasing and variability in care quality creates inefficiencies. In 2007, the Victorian Government (Australia) employed clinical facilitators for three years in eight public hospitals to improve stroke care. Literature on the cost implications of such roles is rare. We report changes in the costs of acute stroke care following implementation of this program.

**Methods:**

Observational controlled before-and-after cohort design. Standardised hospital costing data were compared pre-program (financial year 2006–07) and post-program (2010–11) for all admitted episodes of stroke or transient ischaemic attack (TIA) using ICD-10 discharge codes. Costs in Australian dollars (AUD) were adjusted to a common year 2010. Generalised linear regression models were used for adjusted comparisons.

**Results:**

A 20% increase in stroke and TIA episodes was observed: 2624 pre-program (age > 75 years: 53%) and 3142 post-program (age > 75 years: 51%); largely explained by more TIA admissions (up from 785 to 1072). Average length of stay reduced by 22% (pre-program 7.3 days to post-program 5.7 days, *p* < 0.001). Six hospitals provided cost data. Average per-episode costs decreased by 10% (pre-program AUD7888 to post-program AUD7115). After adjusting for age, sex, stroke type, and hospital, average per-episode costs decreased by 6.1% from pre to post program (*p* = 0.025). When length of stay was additionally adjusted for, these costs increased by 10.8%, indicating a greater mean cost per day (*p* < 0.001).

**Conclusion:**

Cost containment of acute inpatient episodes was observed after the implementation of stroke clinical facilitators, likely associated with the shorter lengths of stay.

**Electronic supplementary material:**

The online version of this article (10.1186/s12913-018-3836-9) contains supplementary material, which is available to authorized users.

## Background

Stroke is a high cost condition, and the majority of stroke-related costs arise from hospitalisation. In the first year, approximately 30% of costs for stroke are attributable to acute hospital care and 30% to inpatient rehabilitation [1, 2]. With the expected increases in the incidence of stroke associated with population ageing, finding more effective and cost-effective methods of providing hospital care for acute stroke within an environment of limited resources is important.

Currently, the most successful and universally applicable intervention for acute stroke in reducing death and disability is organised management in stroke units [3]. Stroke units support greater adherence to evidence-based interventions via a dedicated and specialised interdisciplinary clinical team making them more efficient than general wards [4, 5]. However, in Australia just over half of all patients with stroke receive care in a stroke unit and access to other evidence-based treatments remains variable [6].

Providing a dedicated position for co-ordination or facilitation of uptake of evidence-based care in hospital settings, including the establishment of stroke units, may be one solution to improve stroke care outcomes [7, 8]. Clinical leadership and facilitation, (i.e. where an individual with the appropriate knowledge and skills supports clinicians to change their practice), are cornerstones for enhancing the uptake of evidence [9]. The role of clinical facilitators may include establishing improved systems of communication, providing education and training to upskill staff, reviewing gaps in the provision of evidence-based practice and working with clinicians to improve the quality of care using evidence-based strategies, such as implementing clinical protocols [10, 11]. However, evidence for the effectiveness of clinical facilitators in the field of stroke remains limited [12]. In our previous work we determined that the fixed-term employment of facilitators was effective in positively influencing stroke care in hospitals (e.g. more patients receiving brain imaging within 24 h, greater access to care in a stroke unit and intravenous thrombolysis if an ischaemic stroke) [13]. It remains unknown if there are broader benefits to hospitals associated with facilitator programs. Our aim was to describe the costs of acute care before and after the implementation of the stroke clinical facilitator program in Victoria, Australia.

## Methods

### Setting and context

In Australia, the funding of acute public hospital care is the responsibility of the state government. The Victorian Stroke Clinical Network (VSCN) was established by the Victorian government in 2007. The VSCN comprised an interdisciplinary executive committee of clinicians and government representatives, who meet monthly, to provide board direction for policy and strategies to improve stroke care including advancing recommendations within the Stroke Care Strategy for Victoria. Priorities included improving access to stroke units, thrombolysis, and use of evidence-based care protocols [14]. The VSCN executive endorsed the employment of clinical facilitators for a time-limited period (i.e. 2 to 3 years) in selected priority hospitals based on the results of a national audit, number of patients admitted per year or if in a regional location. The national audit program of acute services is undertaken every two years and is used to monitor adherence to Australian stroke clinical guidelines. At each participating hospital the medical records of up to 40 consecutive patients admitted with a primary diagnosis of stroke are retrospectively audited for patient demographic information, adherence to recommended processes of care and hospital-based patient outcomes [15]. These data were used to identify priority hospitals requiring support to improve adherence to clinical processes of care [13].

**Fig. 1:**
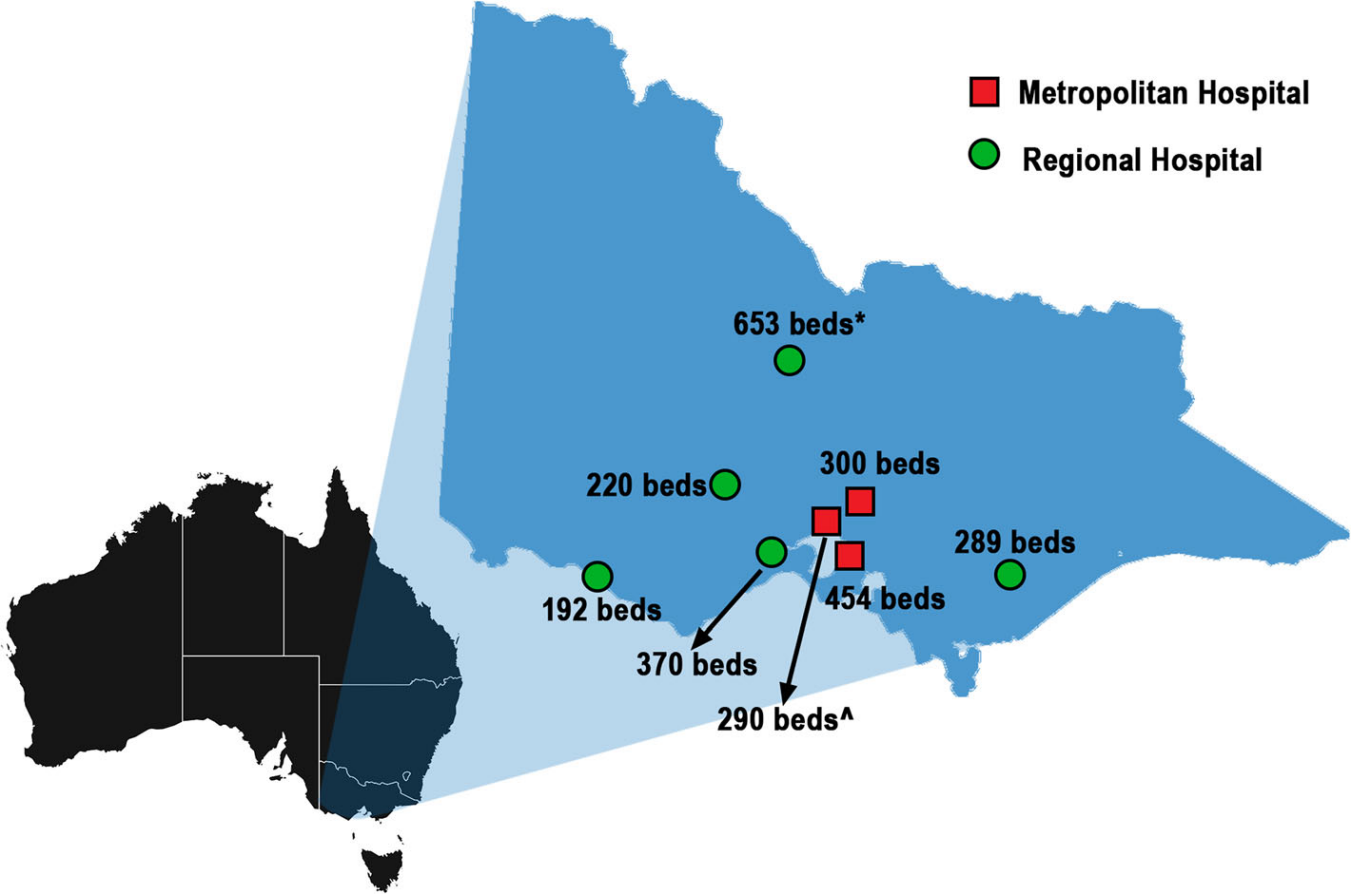
Location of participating hospitals. The map depicted in Fig. 1 was created by the authors by adapting images freely available online. The map of Australia was sourced from: https://pixabay.com/en/australia-continent-geography-map-23497/ and the map of Victoria was sourced from: https://pixabay.com/en/victoria-map-australia-state-23535/ Hospital bed numbers as accessed 10th January 2017 https://www2.health.vic.gov.au/hospitals-and-health-services/public-hospitals-victoria ^ Recent relocation of some services to another campus within health service; * includes on site in-patient rehabilitation beds

From May 2008, these ‘Stroke Network Facilitators’ (herein referred to as Facilitators) commenced in their roles to support the establishment of better systems of care for stroke in the priority hospitals, including establishing stroke units and clinical protocols [12]. These Facilitators had clinical backgrounds in nursing or allied health disciplines and all were employed half time (e.g. 2.5 days per week), with the exception being one large regional hospital where two Facilitators provided fulltime cover [12]. The hospitals included three metropolitan sites within Melbourne (Hospital [H] identification: H4, H6, H8) and five regional sites in other locations within Victoria (Fig. 1). Only three of these hospitals (H1, H2, H5) had a formalised acute stroke unit prior to the Facilitators commencing in their role [12]. The annual cost of employing Facilitators ranged from $45,000 to $60,000 between 2008 and 2011 per Facilitator if working 2.5 days per week (overall ~ $530,000 per year). The cost of employing facilitators was borne by the Victorian Department of Health and not the hospitals. Therefore, these costs were not taken into account as part of the analysis, which was based on anonymised patient-level clinical costing data from hospitals.

### Study design

This was an observational controlled before-and-after cohort design study from the perspective of hospitals. Standardised hospital episode and clinical costing data, which were anonymised and approved for use for a secondary purpose, were supplied by the Victorian Government, and were compared pre-program (financial year [FY] 2006–07) and post-program (FY 2010–11). Eligibility for inclusion in these analyses was based on hospital discharge International Classification of Diseases (ICD) 10 codes for stroke and transient ischaemic attack (TIA). Ischaemic stroke was defined as I63, intracerebral haemorrhage (ICH) as I61, TIA as G45 (excluding G45.4 Transient global amnesia), and Stroke, not specified (for any particular type) as I64. All data received were based on episodes of care and de-identified at an individual patient-level. Therefore, a single person may have had multiple episodes of care for stroke in the dataset. Based on data from the Australian Stroke Clinical Registry this is approximately 4% [16]. Further, we were unable to link episodes and produce estimates for individual patients because a unique personal identifier does not exist in Australia. Only Victorian Admitted Episodes Data linked to clinical costing data were used.

Most, but not all hospitals provide clinical costing data as part of the annual Victorian cost-weights study [17]. All salaries and wages are allocated to different categories including nursing, medical, allied health, emergency care, theatre, pharmacy, pathology, imaging, intensive care or coronary care and other. Medical supplies, pharmaceuticals, pathology equipment, imaging and hotel expenses are also allocated [18]. Indirect costs, including administration, research and training are also attributed to the most appropriate cost category (please see Additional file 1). Six of the eight participating hospitals provided costing data for the present study through the Victorian Government. This was because there were delays in submission of clinical costing data to the Victorian Government, from hospitals H7 and H8, and therefore data from one metropolitan and one regional hospital were unavailable within the time frame of this project. The costs of employing Facilitators were not included.

### Data analysis

The data were analysed using SPSS Statistics Software version 22 (IBM Corp., Armonk, NY). Descriptive statistics, using parametric and non-parametric methods appropriate for the data, were used, as well as generalised linear regression modelling of costs with gamma distributions and log linked for multivariable analyses that adjusted for age, sex, stroke type, and length of stay. Costs were adjusted (i.e. inflated) to a common reference year using the total health price index of 1.0813 for inflating 2006–07 prices to 2010–11 prices [19]. To convert costs to United States dollars please multiply by 1.5 which was the purchasing power parity in 2011 [20].

## Results

**Fig. 2:**
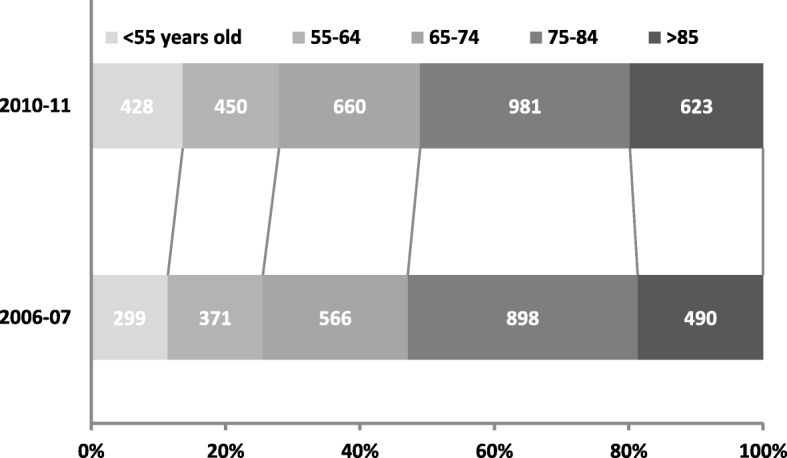
Patient age distributions by study period

The number of episodes of care in 2010–11 increased in all hospitals, on average by 20% since 2006–07 (Table 1). The median number of episodes contributed by the hospitals in 2006–07 was 248 (interquartile range [IQR]: 157–522) and for 2010–11 was 283 (IQR: 208–594). The three hospitals located in the Melbourne metropolitan local government area had a greater number of episodes in both periods with the exception being H2 which is located in the most populous regional area outside of Melbourne (Table 1). Age distributions for the comparison periods are presented in Fig. 2. Overall, there was a 43% increase in the number of patients with stroke or TIA admitted who were aged under 55 years, 21% increase if aged 55–64 years, 17% increase among those aged 65 to 74 years, 9% increase if aged 74–84 years, and 27% increase among those aged 85+ years. Age and sex distributions were also similar between periods for the different stroke types (Additional file 1: Table S1).

**Table 1:** Total episodes of care by acute hospital, stroke type and year

Hospital ID	Ischaemic stroke		Intracerebral haemorrhage		Stroke, not specified		TIA		Total		
Financial Year	2006–07	2010–11	2006–07	2010–11	2006–07	2010–11	2006–07	2010–11	2006–07	2010–11	Δ%
Hospitals in regional Victorian locations											
H1	65	101	21	25	29	27	47	60	162	213	31%
H2	242	269	59	66	42	50	154	184	497	569	14%
H3	44	73	17	21	52	45	29	52	142	191	35%
H7	70	79	15	20	54	51	56	103	195	253	30%
H5	44	45	15	17	26	29	37	55	122	146	20%
Hospitals in Melbourne metropolitan location											
H6	242	407	49	79	134	77	171	226	596	789	32%
H4	153	142	25	31	30	35	92	104	300	312	4%
H8	250	249	72	53	89	79	199	288	610	669	10%
Total	1110	1365	273	312	456	393	785	1072	2624	3142	20%
Total Δ%		23%		14%		−14%		37%		20%	

*H* hospital, *TIA* transient ischaemic attack, Δ% percentage change from 2006 to 07 to 2010–2011, *ID* hospital identification number

Table 2 provides information on the number of episodes, length of stay, total costs and the average cost per episode in each comparison period by stroke type and overall. In 2010–11 there were greater numbers of TIA episodes and fewer strokes classified as type ‘not specified’. Despite the increasing numbers of episodes, the length of stay was significantly reduced over time, by an average of 22% (Table 2). A one day reduction in median length of stay between 2006 and 07 (median 4; IQR 1–9 days) and 2010–11 (median 3; IQR 1–7 days) (*p* < 0.001) was observed. This is mainly explained by shorter lengths of stay for ischaemic stroke (2006–07 median 7; IQR 4–13 days vs. 2010–11 median 5; IQR 3–9 days; p < 0.001) and no change over time was noted in median values for length of stay among patients admitted with ICH (2006–07 median 3; IQR 1–9 days vs. 2010–11 median 3; IQR 1–7 days; *p* = 0.136).

**Table 2:** Summary results by stroke type and year

	Ischaemic stroke		Intracerebral haemorrhage		Stroke, not specified		TIA		Total	
	2006–07	2010–11	2006–07	2010–11	2006–07	2010–11	2006–07	2010–11	2006–07	2010–11
Episodes of care	1110	1365	273	312	456	393	785	1072	2624	3142
Δ%		23%		14%		−14%		37%		20%
Average LOS	10.6	7.9	7.8	6.5	6.4	5.5	2.8	2.6	7.3	5.7
Δ%		*−25%†*		−17%		−13%		−9%		*−22%†*
Costing data^a^										
Total costs (‘000 s)	$8109	$9653	$1586	$1960	$2188	1678	$1605	$2198	$13,488	$15,589
Δ%		19%		24%		−23%		43%		16%
Average per episode cost	$11,093	$9418	$9275	$8447	7245	6454	$3171	$3409	$7888	$7115
Δ%		*−19%†*		−20%		−5%		4%		*−10%†*

*TIA* transient ischaemic attack, Δ% percentage change from 2006 to 07 to 2010–2011. All costs are in 2010 Australian dollars. ^a^Two hospitals are not included since their clinical costing data were not supplied. † t-test *p* < 0.01, all others *p* > 0.1

The analysed episodes reflected a total of 19,037 patient bed-days (total episodes/total number of days) in these acute hospitals in 2006–2007 with a 7% decrease to 17,763 bed-days in 2010–2011. The proportion of beds occupied at any given time by patients with ICH remained stable at 11.2 to 11.4% while the proportion of ischaemic stroke was reduced from 61.9 to 60.8%, and the proportion of patients with TIA increased from 11.6 to 15.5%.

Additional results at an individual hospital level and aggregated by stroke type, where relevant, are provided in (Additional file 1: Tables S1-S4).

### Differences in hospital costs

Overall, the six hospitals with clinical costing data spent AUD13.5 million in the care of these patients in 2006–7 and AUD15.6 million in 2010–11, a 16% increase (Table 2). However, the average per-episode costs decreased by 10% over the 4 years (2006–7 AUD7888 versus 2010–11 AUD7115) (Table 2). After adjusting for patient age, sex, stroke type, and hospital, there was a 6.1% decrease in average per-episode costs between 2006 and 2010 (95% confidence interval 0.9 to 11.4%; *p* = 0.022). However, if length of stay was additionally adjusted for, a 10.8% increase in total cost was observed (95% confidence interval 7.2 to 14.3%, *p* < 0.001), illustrating the increase in cost per day. In other words, the average cost per episode decreased by 6.1%, but the average cost per day increased by 10.8%.

**Fig. 3:**
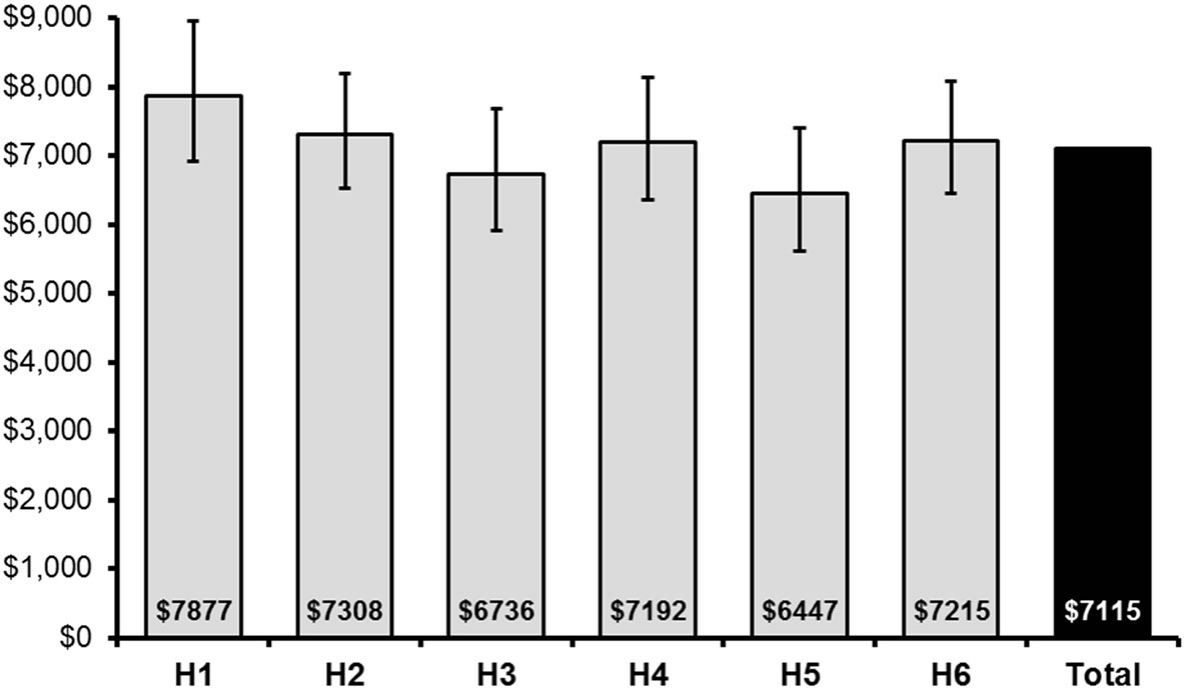
Average costs (in Australian dollars) per episode for 2010–11 adjusted for patient characteristics and length-of- stay. Estimated marginal means and 95% confidence intervals of a generalised linear model with gamma distribution and log link adjusting for age, sex, stroke type, and length of stay. H = hospital. hHospital H4 and H6 were located in the Melbourne metropolitan area (other hospitals are in a regional location within Victoria)

Hospitals with the largest reductions in length of stay (Additional file 1: Table S2) had evidence of the most marked cost containment as shown in Additional file 1: Table S3 (Total in-hospital costs by hospital) and Additional file 1: Table S4 (Average per-episode total inpatient costs by hospital)). After adjusting for length of stay and patient characteristics, there were still differences among the hospitals in 2010–11 between the most expensive (H1: regional site) and least expensive (H3 regional site *p* = 0.005, H5 regional site *p* < 0.001) hospitals in our sample (Fig. 3). Breakdown of mean unadjusted cost per episode in 2010–11 is displayed in Additional file 1: Figure S1. In 2010–11, the main unadjusted cost components overall were nursing (44%), medical (14%), allied health (13%), emergency department (9%), and imaging (8%) (Additional file 1: Figure S2). Differences in mean episode costs for each category were compared between 2006-07 and 2010–11. Allied health, emergency, and imaging costs increased (*p* < 0.01) while nursing, medical and pathology costs decreased (all p < 0.001) over the study period. For all other categories, no significant differences were observed.

## Discussion

Clinical facilitators are recognised as important for improving evidence-based practice as part of organisational redesign or achieving task oriented objectives [10, 11, 13, 21]. To our knowledge, we report for the first time on changes in acute hospital costs associated with investment in Facilitators employed to support improvements in the quality of acute stroke care at priority hospitals. Employed for 3-years, the Facilitators in our study had no clinical caseload as part of their role and were able to establish standardised clinical protocols and care plans, and new dedicated stroke units where these were required [13]. The observed changes in practice included increased access to Stroke Units (pre-facilitator 53%; post 86% *p* < 0.001); intravenous thrombolysis (pre-facilitator 2%; post 9% p < 0.001) and TIA protocols became available in all sites where previously only 50% provided these [22]. Program success factors included sharing of information between the participating hospitals to reduce duplication of effort and the clinical teams having increased capacity to undertake clinical quality improvement projects.

Overall, indications of greater efficiency and capacity to manage more patients with stroke or TIA as inpatients within the context of fewer bed-days being occupied were found. Despite an observed 20% increase in patient episodes, the number of bed-days utilised was fewer, but the average cost per bed-day was larger (2006–07 AUD709 per day; 2010–11 AUD878 per day). Reassuringly we found evidence of cost containment, with the average total inpatient costs per-episode decreasing by 10% over 4 years. The cost containment is explained by reductions in average length of stay, a changing case-mix of patients and the need for hospitals to improve their admission and discharge processes to accommodate greater number of patients. These efforts would have been augmented by the achievements of the Facilitators in creating more streamlined management processes including access to rehabilitation services [13].

Our observed reduction in length of stay averaged 4.1% over the 5 year study period in our priority hospitals with Facilitators, and provides evidence that not all of this reduction is explained by system-wide downward trends. An average annual decrease of 2.1% in length of stay between 2007 and 08 and 2011–12 among acute care public hospitals in Australia, and with same-day separations excluded, has been reported [23]. Several explanations for our findings are possible, including that there were proportionally more TIA episodes than stroke episodes, and that patients with TIA had shorter lengths of stay than patients with stroke.

The absolute average cost of an episode decreased (− 6%). When length of stay was additionally adjusted for, the cost per day increased by ~ 11% highlighting that reductions in lengths of stay do not necessarily result in overall savings. This is because approximately 40% of the variable costs are incurred during the first three days of admission [24]. Reductions in length of stay influence the less expensive days in hospital, and with greater efficiency more patients can be treated, but each bed day is more costly [24]. This was explained by a shift in resource utilisation whereby allied health, emergency, and imaging costs were greater contributors to the overall costs in 2010–11 while nursing, medical and pathology costs, usually associated with ongoing care in hospital decreased for this period when compared with 2006–07 (Additional file 1).

The differences in stroke type patterns may be a consequence of improved documentation and coding, as well as better diagnostic work up [13]. This shift in case-mix may also reflect differences in transfer policies within the geographical areas serviced by these hospitals that have now established stroke units.

There have been very few published studies designed to explore the influence of clinical facilitator roles and hospital costs [25, 26], and none to our knowledge in stroke. In the study by Sakallaris et al. (2000), use of a facilitator early in the process to establish same-day transfer protocols to a cardiac telemetry unit after surgery resulted in cost savings and was achieved without compromising the quality of care (assessed by measuring rates of readmission to the ICU or hospital) [26]. Other related studies include use of clinical pathways. In the most recent systematic review by Rotter and colleagues it was concluded that use of clinical pathways was the most likely explanation for the observed reduced lengths of stay and costs [27]. This is consistent with our findings, which also highlight important efficiency gains despite the increased numbers of admissions. Future research is needed to verify our findings or provide cost-effectiveness evidence against other potential models of facilitation in stroke care. This includes a comparison against stroke coordinator roles that are fully embedded within hospitals [7]. In this way, the value of these alternate options for improving stroke care could more effectively guide policy decision-making.

Strengths of the study include use of comprehensive episode-based costing data for whole financial years obtained using reliably applied clinical costing standards [18] and categorised by resource type and stroke type to provide fuller explanatory information. It has been found that the precision of estimates from the Victorian clinical costing data and generalizability to overall Victorian inpatient care is very good, and that by obtaining costs at an ICD10 rather than diagnostic-related group level, we were more likely to have consistency in classification of patients and costs [28].

Potential limitations include that the evaluation of costs presented here relies on the reliability of ICD-10 discharge codes. However, the method for assigning the nominated ICD-10 discharge codes is unlikely to have changed between these periods. There was also evidence of improved diagnostic classification whereby fewer unspecified strokes were reported in the 2010–11 cohort, but this would not have influenced our overall cost results. In addition, we were unable to follow individual patients and the cost per patient could not be derived since the data were de-identified. It is likely that a small number of individuals (~ 4%) [16] will appear several times in the dataset. Re-hospitalisations may artificially increase the number of episodes and decrease the average costs per episode.

It cannot be assumed that all the benefits observed are fully attributable to the Facilitator program in these hospitals. Nevertheless, the magnitude of the changes observed are unlikely to be fully explained by secular improvements in evidence-based practice. For example, the evidence for the establishment of stroke units has existed since 1993 [29], but poor access to stroke units is still an issue in Australia [30]. In our primary results paper related to this program we reported changes in processes of care in the post-facilitator period that were much greater than the contemporaneous equivalent data from all other hospitals in Australia [13]. In the current study, we also noted differences in the average costs per episode treated between hospitals. This may in part be explained by teaching and research activities allocated as indirect costs [18]. Therefore, the metropolitan hospitals (two of the six that provided cost data) may have greater costs than the regional hospitals. Alternatively, regional hospitals may be required to pay greater staff costs due to more use of Visiting Medical Officers and patient transport services for diagnostic tests. The small number of sites in this analysis means that comparisons between the metropolitan and regional sites are only indicative of possible differences in resource use and should be interpreted with caution.

Our results might also be influenced by some cost shifting from the ambulatory and/or primary care sector to the inpatient hospital sector, or from acute hospitals to sub-acute care settings such as rehabilitation. More rapid discharge to rehabilitation or to home settings with community supports may provide cost savings for acute hospitals, but may increase costs within these other services. Moreover, there are now a range of rapid assessment outpatient options for managing TIA which may be more efficient than care in hospitals [31]. In an ideal situation, a full cost evaluation should include follow-up of individual patients through the whole stroke system of care [2].

## Conclusions

These data provide important new information about changes in patterns of resource use in a sample of Victorian hospitals where there has been a strong focus on improving acute stroke care made possible through Facilitators. The findings are relevant for stroke care internationally, and may also be applicable to other conditions where variation in practice exists. Importantly, the results highlight that health service improvements can be achieved against a background of increased activity and rising costs. Given the paucity of data in this area further research is needed to verify these findings and to understand the longer term sustainability impacts of implementing this type of program.

## Additional file

Additional file 1: **Appendix 1.** Description of data: In this additional file, supplemental Methods are outlined to provide the background to clinical costing methods within hospitals for the state of Victoria (Australia) and additional Results are presented on information at an individual hospital level or aggregated by stroke type in the  supplemental Tables and Figures. (DOC 331 kb)

## Abbreviations

AUD: Australian dollars
Facilitator: Stroke Network Facilitator
H: Hospital
ICD-10: International classification of diseases
ICH: Intracerebral haemorrhage
ID: Identification
IQR: Interquartile range
TIA: Transient ischaemic attack
VSCN: Victorian Stroke Clinical Network
