# Supplementary material for: Changes in acute hospital costs after employing clinical facilitators to improve stroke care in Victoria, Australia
Source: BMC Health Serv Res. 2019 Jan 18;19:41. doi: 10.1186/s12913-018-3836-9 (PMC6337854; doi:10.1186/s12913-018-3836-9)
Supplement: Supplementary file 1 — Appendix 1. Description of data: In this additional file, supplemental Methods are outlined to provide the background to clinical costing methods within hospitals for the state of Victoria (Australia) and additional Results are presented on information at an individual hospital level or aggregated by stroke type in the supplemental Tables and Figures. (DOC 331 kb) [file 12913_2018_3836_MOESM1_ESM.doc]

**APPENDIX 1**

**TITLE: Changes in acute hospital costs after employing clinical facilitators to improve stroke care in Victoria, Australia.**

**AUTHORS & INSTITUTIONS:**

Dominique A Cadilhac^1,2,3^ PhD (dominique.cadilhac@monash.edu)

Helen M Dewey^2,4^PhD (Helen.Dewey@easternhealth.org.au)

Sonia Denisenko^3^ MPH (Sonia.Denisenko@dhhs.vic.gov.au)

Christopher F Bladin^2,4^MD (chris.bladin@unimelb.edu.au)

Atte Meretoja^2,5^ PhD (Atte.Meretoja@hus.fi)

Authors’ affiliation(s):

1. Department of Medicine, School of Clinical Sciences at Monash Health, Monash University, Clayton, Australia
2. Stroke Division, Florey Institute of Neuroscience and Mental Health, University of Melbourne, Heidelberg, Australia
3. Department of Health and Human Services, Melbourne, Australia
4. Eastern Health Clinical School, Monash University, Box Hill, Australia
5. Department of Medicine, Royal Melbourne Hospital, University of Melbourne, Parkville, Australia

**Corresponding author full contact details:**

Professor: Dominique A Cadilhac

Translational Public Health and Evaluation Division, Stroke and Ageing Research, School of Clinical Sciences at Monash Health, Department of Medicine, Monash University, Level 5, Block E Monash Medical Centre, Clayton 3168, Vic Australia,

Phone: +61(3) 8572 2657

**SUPPLEMENTAL METHODS**

Clinical costing data are routinely obtained as part of the annual Victorian cost-weights study.[^1^](#_ENREF_1) Victoria was one of the first states in Australia to adopt a standard chart of accounts that was mandated for public hospitals in the mid-1980s, and has a strong history of standardised clinical costing reporting based on industry approved methods that provide guidance for micro-level costing to ensure the consistent attribution of direct and indirect costs in hospitals.[^2-4^](#_ENREF_2) These cost data are collected using systematic methods endorsed by the Clinical Costing Standards Association of Australia (http://www.ccsaa.com/standards.htm) and conform with the Victorian business rules for clinical costing.[^4^](#_ENREF_4) Briefly, the clinical costing methodology requires that hospitals allocate all their operating expenses from their general ledger in the costing process which must be allocated to patient episodes.[^4^](#_ENREF_4) Operating costs related to teaching and research activities are allocated as indirect costs and to the most appropriate cost area within the costing system, e.g. medical units for training of medical staff. Indirect costs also include items such as medical indemnity costs. All salaries and wages are allocated to different categories including nursing, medical, allied health, emergency care, theatre, pharmacy, pathology, imaging, intensive care or coronary care and other. Medical supplies, pharmaceuticals, pathology equipment, imaging and hotel expenses (including cleaning products, linen and foods services), as well as blood product costs are also allocated.[^4^](#_ENREF_4)

**SUPPLEMENTAL RESULTS**

The following supplemental Tables and Figures of results provide supplemental information at an individual hospital level and aggregated by stroke type, where relevant.

**Table I Age distributions by sex, stroke type** and financial year

|  |  | |  | | **Ischaemic stroke** | | | |  | **Intracerebral haemorrhage** | | | | | | | **Stroke, not specified** | | | | | | | | | **Transient ischaemic attack** | | | | | | | | | |  | | |
| --- | --- | --- | --- | --- | --- | --- | --- | --- | --- | --- | --- | --- | --- | --- | --- | --- | --- | --- | --- | --- | --- | --- | --- | --- | --- | --- | --- | --- | --- | --- | --- | --- | --- | --- | --- | --- | --- | --- |
|  |  | | **2006-07** | | | | **2010-11** | | | **2006-07** | | | | **2010-11** | | | | **2006-07** | | | | **2010-11** | | | | | **2006-07** | | | | **2010-11** | | | | | | |  |
| **Sex** | **Age group (years)** | | **N** | | **%** | | **N** | | **%** | **N** | | **%** | | **N** | | **%** | | **N** | | **%** | | **N** | | **%** | | | **N** | | **%** | | **N** | | | **%** | | | |  |
| **Male** | <55 | 76 | | 13% | | 110 | | 15% | | | 24 | | 15% | | 18 | | | | 11% | | 26 | | 12% | | 23 | | | 11% | | 58 | | 14% | 92 | | 17% | |  | |
|  | 55-59 | 40 | | 7% | | 47 | | 6% | | | 7 | | 5% | | 15 | | | | 9% | | 20 | | 9% | | 13 | | | 6% | | 40 | | 10% | 39 | | 7% | |  | |
|  | 60-64 | 58 | | 10% | | 74 | | 10% | | | 14 | | 9% | | 19 | | | | 12% | | 26 | | 12% | | 20 | | | 10% | | 56 | | 13% | 58 | | 10% | |  | |
|  | 65-69 | 61 | | 10% | | 93 | | 13% | | | 14 | | 9% | | 22 | | | | 14% | | 16 | | 7% | | 22 | | | 11% | | 39 | | 9% | 61 | | 11% | |  | |
|  | 70-74 | 85 | | 15% | | 101 | | 14% | | | 24 | | 15% | | 17 | | | | 11% | | 28 | | 12% | | 26 | | | 13% | | 68 | | 16% | 71 | | 13% | |  | |
|  | 75-79 | 114 | | 20% | | 117 | | 16% | | | 24 | | 15% | | 21 | | | | 13% | | 34 | | 15% | | 29 | | | 14% | | 58 | | 14% | 81 | | 15% | |  | |
|  | 80-84 | 78 | | 13% | | 110 | | 15% | | | 27 | | 17% | | 22 | | | | 14% | | 38 | | 17% | | 35 | | | 17% | | 58 | | 14% | 72 | | 13% | |  | |
|  | 85+ | 71 | | 12% | | 92 | | 12% | | | 21 | | 14% | | 27 | | | | 17% | | 38 | | 17% | | 36 | | | 18% | | 38 | | 9% | 81 | | 15% | |  | |
|  | ***subtotal*** | ***583*** | |  | | ***744*** | |  | | | ***155*** | |  | | ***161*** | | | |  | | ***226*** | |  | | ***204*** | | |  | | ***415*** | |  | ***555*** | |  | |  | |
| **Female** | <55 | 37 | | 7% | | 68 | | 11% | | | 12 | | 10% | | 13 | | | | 9% | | 14 | | 6% | | 15 | | | 8% | | 52 | | 14% | 89 | | 17% | |  | |
|  | 55-59 | 12 | | 2% | | 30 | | 5% | | | 3 | | 3% | | 7 | | | | 5% | | 7 | | 3% | | 6 | | | 3% | | 22 | | 6% | 32 | | 6% | |  | |
|  | 60-64 | 34 | | 6% | | 37 | | 6% | | | 5 | | 4% | | 7 | | | | 5% | | 11 | | 5% | | 9 | | | 5% | | 16 | | 4% | 37 | | 7% | |  | |
|  | 65-69 | 43 | | 8% | | 41 | | 7% | | | 4 | | 3% | | 10 | | | | 7% | | 9 | | 4% | | 11 | | | 6% | | 38 | | 10% | 21 | | 4% | |  | |
|  | 70-74 | 65 | | 12% | | 68 | | 11% | | | 6 | | 5% | | 16 | | | | 11% | | 25 | | 11% | | 20 | | | 11% | | 41 | | 11% | 60 | | 12% | |  | |
|  | 75-79 | 96 | | 18% | | 80 | | 13% | | | 20 | | 17% | | 23 | | | | 15% | | 44 | | 19% | | 23 | | | 12% | | 57 | | 15% | 61 | | 12% | |  | |
|  | 80-84 | 111 | | 21% | | 124 | | 20% | | | 29 | | 25% | | 33 | | | | 22% | | 45 | | 20% | | 47 | | | 25% | | 65 | | 18% | 103 | | 20% | |  | |
|  | 85+ | 129 | | 24% | | 173 | | 28% | | | 39 | | 33% | | 42 | | | | 28% | | 75 | | 33% | | 58 | | | 31% | | 79 | | 21% | 114 | | 22% | |  | |
|  | ***subtotal*** | ***527*** | |  | | ***621*** | |  | | | ***118*** | |  | | ***151*** | | | |  | | ***230*** | |  | | ***189*** | | |  | | ***370*** | |  | ***517*** | |  | |  | |
| **Total** |  | **1110** | |  | | **1365** | |  | | | **273** | |  | | **312** | | | |  | | **456** | |  | | **393** | | |  | | **785** | |  | **1072** | |  | |  | |

**Table II Average length of stay in days by hospital, stroke type and year**

| ID | Ischaemic stroke | | Intracerebral haemorrhage | | Stroke, not specified | | TIA | | Total | | |
| --- | --- | --- | --- | --- | --- | --- | --- | --- | --- | --- | --- |
|  | 2006-07 | 2010-11 | 2006-07 | 2010-11 | 2006-07 | 2010-11 | 2006-07 | 2010-11 | 2006-07 | 2010-11 | Δ% |
| ***Hospitals in regional Victorian locations*** | | | | | | | | |  |  |  |
| H1 | 8.0 | 6.6 | 5.3 | 9.0 | 6.0 | 5.1 | 3.4 | 2.4 | 6.0 | 5.5 | -8% |
| H2 | 13.6 | 8.4 | 11.3 | 6.6 | 8.3 | 6.7 | 3.5 | 3.3 | 9.7 | 6.4 | -34% |
| H3 | 10.4 | 12.3 | 7.2 | 5.7 | 7.8 | 6.3 | 3.8 | 5.2 | 7.7 | 8.2 | 6% |
| H7 | 8.0 | 5.6 | 2.5 | 3.0 | 4.7 | 3.7 | 2.3 | 2.8 | 5.0 | 3.9 | -23% |
| H5 | 7.6 | 5.0 | 8.3 | 5.6 | 9.6 | 7.8 | 2.8 | 2.7 | 6.6 | 4.7 | -29% |
| ***Hospitals in Melbourne metropolitan location*** | | | | | | | | |  |  |  |
| H4 | 8.6 | 6.5 | 4.5 | 4.6 | 3.7 | 4.7 | 2.5 | 2.0 | 5.9 | 4.6 | -22% |
| H6 | 13.8 | 8.4 | 10.3 | 8.4 | 7.7 | 5.1 | 2.8 | 2.3 | 9.0 | 6.3 | -30% |
| H8 | 7.8 | 7.9 | 6.2 | 5.4 | 3.7 | 5.7 | 2.4 | 2.0 | 5.2 | 4.9 | -7% |
| Total | 10.6 | 7.9 | 7.8 | 6.5 | 6.4 | 5.5 | 2.8 | 2.6 | 7.3 | 5.7 | -22% |
| Total Δ% |  | -25% |  | -17% |  | -13% |  | -9% |  | -22% |  |

H=hospital; TIA=transient ischaemic attack; Δ%=percentage change from 2006-07 to 2010-2011; ID: identification code for hospital.

**Table III Total in-hospital costs (AUD thousands) by hospital, stroke type and year**

|  | Ischaemic stroke | | Intracerebral haemorrhage | | Stroke, not specified | | TIA | | Total | | |
| --- | --- | --- | --- | --- | --- | --- | --- | --- | --- | --- | --- |
| ID | 2006-07 | 2010-11 | 2006-07 | 2010-11 | 2006-07 | 2010-11 | 2006-07 | 2010-11 | 2006-07 | 2010-11 | Δ% |
| ***Hospitals in regional Victorian locations*** | | | | | | | | |  |  |  |
| H1 | 510 | 833 | 113 | 316 | 175 | 158 | 180 | 213 | 978 | 1520 | 55% |
| H2 | 3160 | 2761 | 671 | 466 | 337 | 355 | 537 | 706 | 4705 | 4288 | -9% |
| H3 | 245 | 555 | 97 | 133 | 349 | 229 | 87 | 158 | 778 | 1075 | 38% |
| H5 | 244 | 241 | 91 | 105 | 168 | 232 | 84 | 144 | 587 | 722 | 23% |
| ***Hospitals in Melbourne metropolitan location*** | | | | | | | | |  |  |  |
| H4 | 955 | 1064 | 52 | 129 | 81 | 192 | 211 | 315 | 1299 | 1701 | 31% |
| H6 | 2997 | 4199 | 562 | 809 | 1079 | 512 | 504 | 762 | 5143 | 6282 | 22% |
| Total | 8109 | 9653 | 1586 | 1960 | 2188 | 1678 | 1605 | 2298 | 13488 | 15589 | 16% |
| Total Δ% |  | 19% |  | 24% |  | -23% |  | 43% |  | 16% |  |

H=hospital; H7 and H8 not included due to data not being available. All costs are in 2010 Australian dollars (AUD); TIA=transient ischaemic attack; Δ%=percentage change from 2006-07 to 2010-2011; ID: identification code for hospital.

**Table IV Average per episode total inpatient costs (AUD) by hospital, stroke type, and year**

|  | **Ischaemic stroke** | | **Intracerebral haemorrhage** | | **Stroke, not specified** | | **TIA** | | **Total** | | |
| --- | --- | --- | --- | --- | --- | --- | --- | --- | --- | --- | --- |
| ID | 2006-07 | 2010-11 | 2006-07 | 2010-11 | 2006-07 | 2010-11 | 2006-07 | 2010-11 | 2006-07 | 2010-11 | Δ% |
| ***Hospitals in regional Victorian locations*** | | | | | | | | |  |  |  |
| H1 | 7847 | 8252 | 5397 | 12638 | 6019 | 5838 | 3826 | 3549 | 6035 | 7136 | 18% |
| H2 | 13056 | 10262 | 11565 | 7065 | 8019 | 7096 | 3490 | 3859 | 9485 | 7549 | -20% |
| H3 | 6610 | 8537 | 6454 | 6667 | 6839 | 5213 | 3012 | 3156 | 5890 | 6008 | 2% |
| H5 | 5534 | 5357 | 6074 | 6194 | 6704 | 7997 | 2282 | 2620 | 4848 | 4948 | 2% |
| ***Hospitals in Melbourne metropolitan location*** | | | | | | | | |  |  |  |
| H4 | 9270 | 7712 | 3998 | 5177 | 3845 | 5825 | 3106 | 3151 | 6336 | 5746 | -9% |
| H6 | 12486 | 10317 | 11469 | 10246 | 8056 | 6651 | 2949 | 3371 | 8657 | 7962 | -8% |
| Total | 11093 | 9418 | 9275 | 8447 | 7245 | 6454 | 3171 | 3409 | 7888 | 7115 | -10% |
| Total Δ% |  | -15% |  | -9% |  | -11% |  | 8% |  | -10% |  |

H=hospital; H7 and H8 not included due to data not being available. All costs valued in 2010 Australian dollars (AUD); TIA=transient ischaemic attack; Δ%=percentage change from 2006-07 to 2010-2011; ID: identification code for hospital.

Figure I Average unadjusted cost (Australian dollars [AUD]) per episode for 2010-11 broken down to cost components

Hospital H4 and H6 located in metropolitan Melbourne area (other hospitals are in a regional location within Victoria).


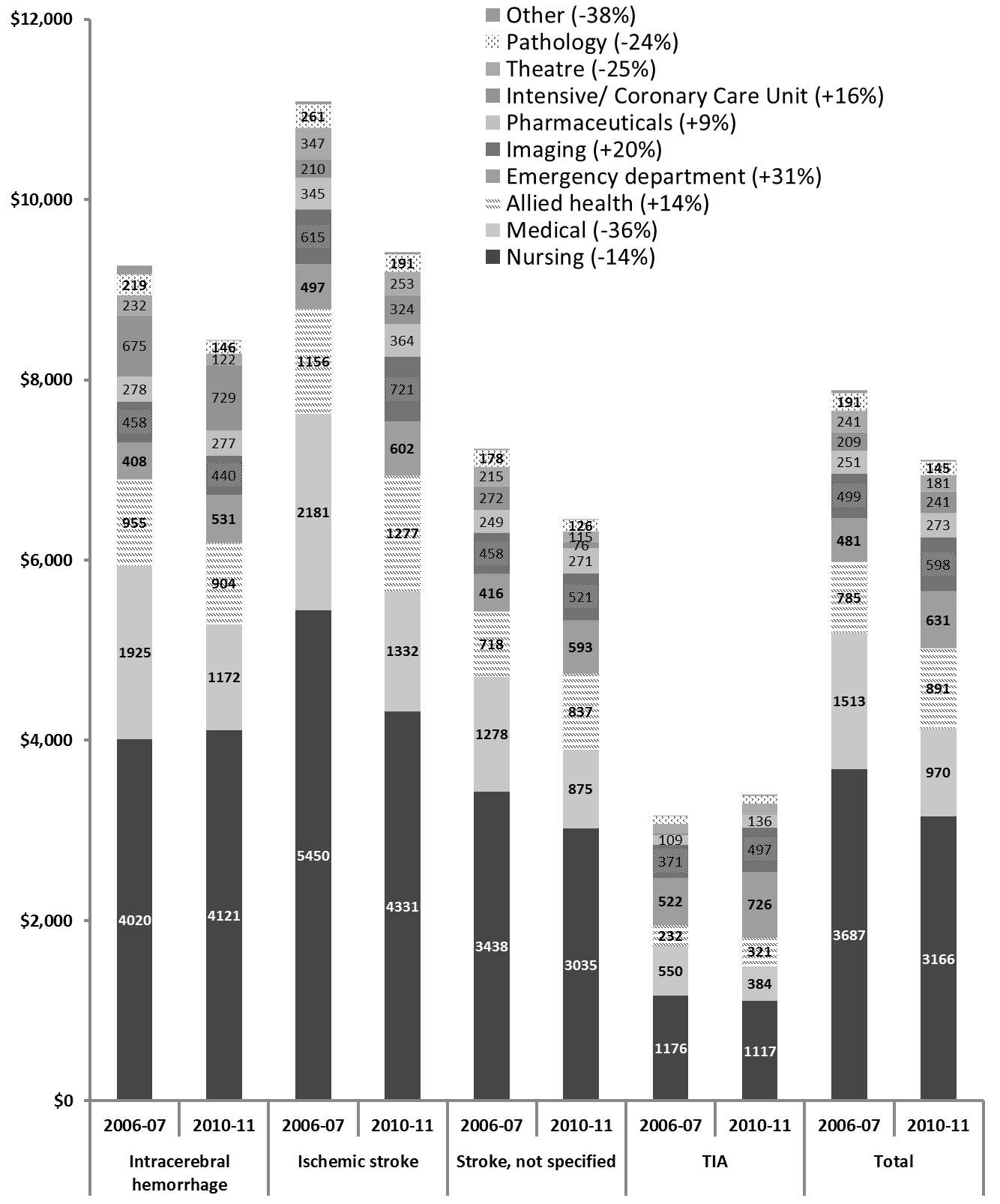


Figure II Total unadjusted average cost (Australian dollars [AUD]) per episode according to individual cost categories. All costs valued in 2010-11 common year.

TIA: transient ischaemic attack

**SUPPLEMENTAL REFERENCES**

1. Victorian Department of Health. Victorian health services policy and funding guidelines 2010-11 – Technical guidelines. 2010:98.

2. Jackson T. Using computerised patient-level costing data for setting DRG weights: the Victorian (Australia) cost weight studies. *Health Policy*. 2001;56:149-63.

3. Australian Goverment Department of Health and Ageing. Australian Hospital Patient Costing Standards version 2.0. 2011:92.

4. Victorian Department of Health. Victorian Cost Data Collection: Business Rules for Reporting 2010-11 Cost Data Version 3.6. 2012:26.
